# Effect of Comprehensive Dental Rehabilitation on Growth Parameters in Pediatric Patients with Severe Early Childhood Caries

**DOI:** 10.5005/jp-journals-10005-1326

**Published:** 2016-04-22

**Authors:** Jayna Sachdev, Kalpana Bansal, Radhika Chopra

**Affiliations:** 1 Ex-Postgraduate Student, Department of Pedodontics and Preventive Dentistry, SGT Dental College, Hospital and Research Institute, Gurgaon Haryana, India; 2 Professor and Head, Department of Pedodontics and Preventive Dentistry, SGT Dental College, Hospital and Research Institute, Gurgaon Haryana, India; 3 Reader, Department of Pedodontics and Preventive Dentistry, ITS Centre for Dental Studies and Research, Ghaziabad, Uttar Pradesh, India

**Keywords:** Catch-up growth, Height, Rehabilitation, S-ECC, Weight.

## Abstract

**Background:** Children who have severe early childhood caries (S-ECC) weigh significantly less than caries-free children. The association between S-ECC and weight suggests that its timely treatment at early stages may preserve general health, in addition to preventing pain and infection.

**Objective:** This study was conducted to evaluate whether children with untreated S-ECC had lower weight and height as compared with children with low caries and to evaluate whether full mouth rehabilitation of children with S-ECC resulted in the phenomenon of catch-up growth.

**Materials and methods:** The weight and height of children with noncontributory medical histories and S-ECC (3-6 years) were compared with caries-free children, before and 6 months after full mouth dental rehabilitation.

**Results:** Prior to dental rehabilitation, children with S-ECC had significantly less weight and height than their comparison counterparts (p < 0.001). Following therapeutic intervention, the test group children exhibited catch-up growth in relation to weight, as there was no significant difference in the body weight of the test and control groups (p = 0.171).

**Conclusion:** Comprehensive full mouth rehabilitation of children with S-ECC results in catch-up growth, thus improving the overall health of the child.

**How to cite this article:** Sachdev J, Bansal K, Chopra R. Effect of Comprehensive Dental Rehabilitation on Growth Parameters in Pediatric Patients with Severe Early Childhood Caries. Int J Clin Pediatr Dent 2016;9(1):15-20.

## INTRODUCTION

Oral health is an essential component of total health and well-being. Despite increase in the availability of caries preventive measures, such as fluoride dentifrices and mouth washes, dental caries still continues to be highly prevalent in the early childhood period.^1,2^ Early childhood caries (ECC) has been defined as “the presence of one or more decayed (noncavitated or cavitated lesions), missing (due to caries), or filled tooth surfaces” in any primary tooth in a child under the age of 6 years.^3^ Severe early childhood caries (S-ECC) is defined as any sign of smooth surface caries in children under 3 years of age. From ages 3 through 5 years, one or more cavitated, missing (due to caries) or filled smooth surfaces in primary maxillary anterior teeth or a decayed, missing or filled score of ≥4 (age 3 years), ≥5 (age 4 years) or ≥6 (age 5 years) surfaces constitutes S-ECC.^3^

Dental caries continues to be a major health problem in the developing nations because of the lack of education and awareness and poor socioeconomic status.^4-6^ Socioeconomic status influences the nutrition and access for health care services.^7^ In the developing nations children suffer from a dual risk of malnutrition, with obesity in those living in urban areas and undernutrition in children from rural and slum areas.^8^ Recently, the United Nations International Children’s Emergency Fund reported that about 146 million children below 5 years of age were underweight.^9^

The potential impact of S-ECC on the general health and development has been widely reported in the literature. On a population basis, it has been observed that with advancing age, and presumably increasing severity of ECC, there was a deceleration of weight gain such that older children with nursing caries were more likely to be represented by lower weight percentile categories.^10^ Furthermore, dental interventions in ECC children had a significant positive impact on parental ratings of their overall oral health and physical, mental and social functioning.^11^ The greatest improvement was noted in pain experience followed by improved abilities to eat and sleep along with parental satisfaction.^12^ The phenomenon of catch-up growth has been reported to occur in children, whose growth had been slowed down by malnutrition following complete dental intervention.^10^

The prevalence of ECC in children in the rural regions of Haryana is very high,^13,14^ hence the present study was planned, firstly, to determine if young children with S-ECC with nonsignificant medical histories (absence of systemic illness) that receive complete dental treatment have less weight and height preoperatively compared with age- and sex-matched low caries children and, secondly, whether full mouth rehabilitation of these patients can bring their weight and height measures comparable to those of low caries children and they demonstrate a phenomenon of catch-up growth by growth velocity calculation.

## MATERIALS AND METHODS

This case-control follow-up study was carried out in the Department of Pedodontics and Preventive Dentistry, Shree Guru Gobind Singh Tricentenary (SGT) Dental College, Gurgaon, Haryana, India. The study was reviewed and approved by the Ethical Committee of SGT Dental College, Gurgaon. Informed consent was taken from parents or legal guardians prior to patient enrolment in the study.

A total of 80 children within the age range of 3-6 years from low socioeconomic status families, who visited the Department of Pedodontics, were included in the study. The children who were physically and mentally compromised, were on medications and had undergone prior invasive dental treatment (extractions or restorations) were excluded from the study. The included children were divided into two equal groups: test and control groups. The test group (n = 40) included children with minimum of four severely decayed teeth with at least two teeth that had pulpal involvement. Children having less than or equal to three enamel or dentinal carious lesions without pulpal involvement formed the control group (n = 40). The pulpal involvement was detected clinically and confirmed radiographically. Both the groups had similarly matched age, gender and socioeconomic status.

Baseline data included recording of decayed, extracted and filled teeth (deft index) followed by the anthropometric measurements height (Ht) in centimeters, weight (Wt) in kilograms. Caries status of the child was recorded using deft index.^15^ Weight and height measurements were taken for each child dressed in light clothes and no shoes. Height was measured using a wall-mounted stadiometer and weight using standard weighing machine after calibration.^5^

After recording the preliminary data, complete dental rehabilitation of S-ECC children was performed according to individual needs. The complete dental treatment included restorations, pulp therapies, extractions if required followed by space management in routine dental setting over multiple visits. The number of visits depended upon the caries status and the child’s cooperation. The child and parents were also given educational preventive counseling for dental health.

After complete dental rehabilitation, the children were recalled for a follow-up visit after a period of 6 months. The anthropometric measurements were repeated at the 6-month recall visit following the completed dental treatment. The growth velocity, a mathematically derived measurement, by convention, is expressed in terms of growth per 6-month period.^6^ It was calculated as follows^16^:

Growth velocity per month for weight (weight/height velocity) can be calculated as:

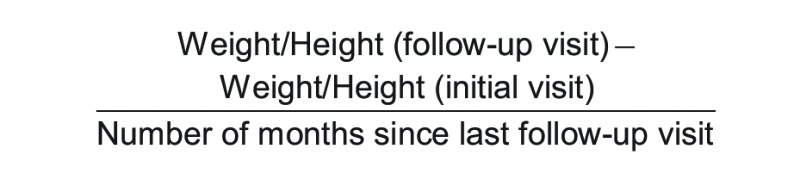



## STATISTICAL ANALYSIS

The statistical analysis was done using Statistical Package for the Social Sciences (SPSS) version 19.0 computer software (SPSS Inc, Chicago, IL, USA). One sample t-test was used to analyze the differences in height and weight of the test group preoperatively as compared with age- and sex-matched caries-free children. The growth velocities in the test and control groups were compared using independent sample t-test. Associations and differences were considered significant when the p value was <0.05.

## RESULTS

A total of 80 subjects (40 test and 40 control) meeting the inclusion criteria were enrolled in the study. Both the groups consisted of 30 boys and 10 girls each. Of them, 15 were excluded from the test group due to incomplete treatment or loss of follow-up; hence, there were a total of 25 patients in the test group during follow-up. We included 25 patients randomly from the control group for statistical evaluation. The test and control subjects were still age- and sex-matched after the sample was reduced. The mean age in both the groups was 4.57 years. The follow-up length for the test group ranged from a minimum of 6 months, extending up to 8 months with an average being 6.6 months, whereas for the control group the follow-up length was 6.5 months.

### Comparison between Mean Values of Baseline Anthropometric Measurements of Control and Test Groups

At, baseline the test group children (n = 40) had a weight of 13.96 ± 2.20 kg as compared with 15.80 ± 1.37 kg in the control group (n = 40), the difference being statistically significant (p < 0.001). Similarly, a statistically significant difference was seen in relation to height (p = 0.002), the test group (n=40) children having a mean height of 101.07 ± 6.71 cm with the control group (n = 40) having a mean height of 105.43 ± 5.43 cm. Also, a statistically significant difference was seen in the deft status (p < 0.001) among the test and the control groups as depicted in Table 1.

For 50 children who were available for follow-up, the baseline statistics were:

- Test group (n = 25): mean weight was 14.24 ± 2.30 kg and mean height was 101.68 ± 7.40 cm.
- Control group (n=25): mean weight was 15.76 ± 1.42 kg and mean height was 106.93 ± 5.20 cm.

The difference between test and control groups at baseline was statistically significant (p < 0.001).

### Comparison between Mean Values of Follow-up Anthropometric Measurements of Control and Test Groups

**Graph 1:**
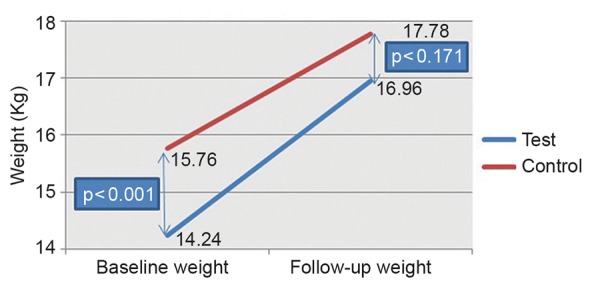
Comparison between baseline and follow-up weight in the test and control groups

From Table 2, it is evident that no statistically significant difference (p=0.171) was seen in relation to weight between test (16.96 kg) and control groups (17.78 kg) at the follow-up visit, as compared with the baseline values, depicting a phenomenon of catch-up growth in the test group subjects (Graph 1). However, there was a statistically significant difference (p = 0.003) between the follow-up height of the test (106.99 cm) and control groups (112.81 cm), indicating no catch-up growth in terms of height. A statistically significant difference was also seen between the difference in weight (weight at follow-up minus weight at baseline) when comparing the test (2.72 kg) and the control groups (2.02 kg) with a p-value of <0.001, indicating more weight gain in the test subjects than in the control subjects. However, no statistically significant difference was seen between the height difference (p = 0.258) and also in relation to age (p = 0.302) among both the groups.

**Table 1:** **Table 1:** Comparison between mean values of baseline anthropometric measurements of control and test groups

		*Group*		*N*		*Mean*		*Standard* *deviation*		*t-* *value*		*P-* *value*	
Age (years)		Test		40		4.48		0.68		1.28		0.204
		Control		40		4.65		0.53					
Height (cm)		Test		40		101.07		6.71		3.2		0.002	
		Control		40		105.43		5.43					
Weight (kg)		Test		40		13.96		2.2		4.49		<0.001	
		Control		40		15.8		1.37					
Deft (N)		Test		40		7.95		2.22		19.9		<0.001	
		Control		40		0.63		0.7					

**Table 2:** **Table 2:** Comparison between mean values of follow-up anthropometric measurements of control and test groups

		*Group*		*N*		*Mean*		*Standard* *deviation*		*t-* *value*		*P-* *value*	
Height (cm)		Test		25		106.99		7.56		3.185		0.003	
		Control		25		112.81		5.15					
Weight (kg)		Test		25		16.96		2.48		1.391		0.171	
		Control		25		17.78		1.59					
Weight		Test		25		2.72		0.63		3.761		<0.001	
difference*		Control		25		2.02		0.68					
Height		Test		25		5.31		1.52		1.145		0.258	
difference*		Control		25		5.88		1.98					
Age (years)		Test		25		4.4		0.76		1.044		0.302	
		Control		25		4.6		0.58					

*Weight and height difference = Follow-up value - Baseline value

### Comparison between the Growth Velocity and Growth Velocity Percentage in the Test and Control Groups in Terms of Height and Weight

The weight velocity for the test group came out to be 0.41 kg/month and for the control group to be 0.31 kg/ month, demonstrating a highly statistically significant difference (p = 0.001), thus depicting the phenomenon of catch-up growth in the test group (Table 3). In terms of weight velocity percentage (postoperative weight minus preoperative weight divided by initial weight and then multiplied by 100) also, statistically significant difference was obtained (p = 0). However, no statistically significant difference was seen in terms of height velocity (p = 0.313) and height velocity percentage (postoperative height minus preoperative height divided by initial height and then multiplied by 100) (p = 0.576) comparing the test and control groups, showing that the test children could not catch up with the control children when height was taken as a growth measure.

## DISCUSSION

Early childhood caries has been recognized as an infectious and transmissible childhood disease with long-term growth and developmental implications.^17^ The concept that dental disease and child’s body weight may be related was raised as early as 1982, when a retrospective case-note study that examined the body weights of children coming for tooth extractions under general anesthesia were compared with children coming for routine dental care,^18^ and subsequent studies have suggested that treatment of caries may lead to improvement in weight, gain^10,19^ at least in children whose weight is below average.^20^

**Table 3:** **Table 3:** Comparison between the growth velocity and growth velocity percentage in the test and control groups in terms of height and weight

*Growth* *velocity*		*Group*		*N*		*Mean*		*Standard* *deviation*		*t-* *value*		*P-* *value*	
Weight		Test		25		0.41		0.09		3.67		0.001	
velocity		Control		25		0.31		0.11					
Weight		Test		25		19.49		5.03		4.82		0	
velocity %		Control		25		12.91		4.61					
Height		Test		25		0.82		0.28		–1.02		0.313	
velocity		Control		25		0.9		0.3					
Height		Test		25		5.25		1.59		–0.56		0.576	
velocity %		Control		25		5.53		1.92					

The above-mentioned association between dental caries and children’s weights is based on the fact that the manifestations of untreated ECC may go beyond pain and infection and the condition may also affect the general health.^10,21^ Consequences of untreated ECC include a higher risk of new carious lesions in both the primary and permanent dentitions, hospitalizations and emergency room visits, increased treatment costs, risk for delayed physical growth and development, loss of school days and increased days with restricted activity, diminished ability to learn and diminished oral health-related quality of life.^3^ Dental treatment also makes a very significant difference to the psychological and social aspects of the child’s life.^12,22-25^ These improvements include less pain and improved abilities to eat and sleep.

The results of our study show a significant correlation between untreated dental caries and decreased body weight. The test group subjects weighed significantly less (p < 0.001) than the control group at baseline. Also, there was a highly statistically significant difference (p = 0.002) between the height of test and control groups, the test subjects being shorter than control subjects at baseline. Our findings corroborated with those of Miller et al^18^ who obtained similar results where 1,105 children who required extractions were significantly lighter than the weights of 527 children who did not require dental extractions. Acs et al^21^ showed that 3-year-old with nursing caries with at least one pulpally involved tooth weighed about 1 kg less than control children without nursing caries. Ayhan et al^26^ also concluded that ECC children had lower mean weight than those of caries-free comparisons, thereby suggesting that ECC children had inadequate caloric consumption. Gaur and Nayak^5^ also compared the height and weight of children with S-ECC and caries-free children. They found that mean height and weight of the S-ECC group was less as compared with that of the caries-free control group.

There are at least three highly plausible mechanisms for how dental caries may be associated with underweight and poor growth in young children. First, untreated caries and associated infection can cause pain and discomfort and reduce intake of foods because eating is painful.^10,27^ Second, severe caries can affect children’s quality of life and thereby growth. Impacts include pain, irritability and disturbed sleeping habits.^22,27^ Disturbed sleep may affect glucosteroid production and growth. Untreated dental caries significantly impacts on the quality of life of children and their dietary intake.^22,24^ The consequences of high caries levels also include a higher risk of hospitalizations and emergency dental visits, increased days with restricted activity and absence from school and a diminished ability to learn.^27^ A third possible mechanism of how untreated severe caries with pulpitis affects growth is that chronic inflammation from pulpitis and chronic dental abscesses affect growth via chronic inflammation affecting metabolic pathways where cytokines affect erythropoiesis. For example, interleukin-1 (IL-1), which has a wide variety of actions in inflammation, can induce inhibition of erythropoiesis. This suppression of hemoglobin can lead to anemia of chronic disease as a result of depressed erythrocyte production in the bone marrow.^27,28^

Growth velocity is expressed in terms of growth per 6-month period. Growth velocity is studied to show how much growth varies at various ages, what patterns of growth are revealed and what they can tell us about the growth of pediatric patients. For velocity information, a longitudinal method is necessary by which the same individuals are measured at specific ages over a period of time. Thus, incremental measures are sought. The improved velocity rates are depictive of periods of catch-up growth.^29^

Healthy children establish and follow a stable velocity growth curve for weight and height. Various insults, including nutritional deficiencies, may deflect children from their normal growth curve temporarily. With correction of these conditions, children will usually exhibit “catch-up growth”, rising initially above before falling back onto the established growth curve.^23^

In terms of growth velocity, the difference was highly statistically significant (p = 0.001) in terms of weight velocity and weight velocity percentage (p = 0) in this study, thus depicting the phenomenon of catch-up growth in the test group. However, no statistically significant difference was seen in terms of height velocity (p = 0.313) and height velocity percentage (p = 0.576) comparing the test and control groups.

After dental rehabilitation, the test group subjects showed a significant increase in weight in this study, which is similar to that reported in earlier studies.^5,10,20^ The catch-up growth observed in the test patients following complete dental rehabilitation suggests that untreated ECC was responsible for the age-adjusted weight differences between the two groups. Following complete dental rehabilitation of children with S-ECC, there was no longer any statistical difference (p = 0.171) in their age-adjusted weights when compared with the control group children.

In our, study no statistically significant difference was seen in terms of height velocity (p = 0.313) and height velocity percentage (p = 0.576) comparing the test and control groups. Also, there was a statistically significant difference (p = 0.003) between the follow-up height of the test (106.99 cm) and control groups (112.81 cm), depicting no catch-up growth in terms of height. This can be attributed to the fact that linear growth does not begin until the child has achieved at least 85% of the expected weight for length (Walker and Golden, 1988). Also, Golden concluded that weight gain precedes height gain,^30^ thus explaining no catch-up growth in terms of height. Further studies incorporating a larger sample size and longitudinal evaluation are required to confirm the results of this study and to evaluate whether oral health has an impact on general health and well-being of the child.

## CONCLUSION

Thus, it can be concluded that children with S-ECC have less weight and height as compared with other children with low caries. After dental rehabilitation, there was a significant improvement in the weight and height of S-ECC group children. The mean values of growth parameters were still higher in the control group as compared with the S-ECC group; however, the difference in values was less as compared with baseline. Overall intergroup comparison after dental rehabilitation showed that S-ECC group no longer differed from the controls in relation to weight.
